# Resistive switching transparent SnO_2_ thin film sensitive to light and humidity

**DOI:** 10.1038/s41598-023-45790-0

**Published:** 2023-11-16

**Authors:** Asiyeh Kalateh, Ali Jalali, Mohammad Javad Kamali Ashtiani, Mohammad Mohammadimasoudi, Hajieh Bastami, Majid Mohseni

**Affiliations:** 1 Electrical Engineering Department, Shahid Beheshti University, Tehran, Iran; 2 Physics Department, Shahid Beheshti University, Tehran, Iran; 3 Nano-Bio-Photonics Lab, Faculty of New Sciences and Technologies, University of Tehran, Tehran, Iran; 4 Department of Materials and Metallurgical Engineering, Technical and Vocational University, Tehran, Iran

**Keywords:** Electronic properties and materials, Electronic devices

## Abstract

Designing and manufacturing memristor devices with simple and less complicated methods is highly promising for their future development. Here, an Ag/SnO_2_/FTO(F-SnO_2_) structure is used through the deposition of the SnO_2_ layer attained by its sol via the air-brush method on an FTO substrate. This structure was investigated in terms of the memristive characteristics. The negative differential resistance (NDR) effect was observed in environment humidity conditions. In this structure, valance change memory and electrometalization change memory mechanisms cause the current peak in the NDR region by forming an OH^−^ conductive filament. In addition, the photoconductivity effect was found under light illumination and this structure shows the positive photoconductance effect by increasing the conductivity. Memristivity was examined for up to 100 cycles and significant stability was observed as a valuable advantage for neuromorphic computing. Our study conveys a growth mechanism of an optical memristor that is sensitive to light and humidity suitable for sensing applications.

## Introduction

Memristors have gained significant attention as a promising technology for the development of nonvolatile memories^1^ and neuromorphic computing^2^. Their structure is simple and has low power consumption for application in fast reading and writing^3–5^. They show potential scalability, high density, low cost, and compatibility with complementary metal oxide semiconductor (CMOS) technology^6–10^.

The operation of a memristor is based on the migration of ions. There are several primary mechanisms in memristors such as valance change memory (VCM), electrochemical metallization memory (ECM), phase change memory (PCM), and thermally change memory (TCM)^2^. Among them, the common mechanisms for a simple metal/insulator/metal (MIM) structure are the ECM^11^ and VCM^12^. In ECM memristors, the transfer of metal cations through the insulating layer is achieved by oxidizing the active metal in the top electrode (TE) upon applying a bias voltage^12–14^.

Transition metal oxides (TMOs) are one of the most appealing materials utilized in resistive switching devices^15^. Among them, a variety of metal oxides, including (e.g., TiO_2_^16,17^, HfO_2_^18^, Ta_2_O_5_^19^, ZnO^20^, ZrO_2_^21^) and transparent conducting oxides, such as SnO_2_^22^, and indium tin oxide (ITO)^22,23^ have also been investigated as switching layers in Resistive Random-Access Memories (RRAMs). In these TMOs, a combination of features like high thermodynamic stability, large band gap, and high dielectric coefficient made them intriguing structures for use in memristor devices^21^.

Among metal oxides, SnO_2_ is a semiconducting material that has attracted attention due to its unique properties in the field of oxide electronics. One of the key features of this semiconductor is its wide bandgap of approximately 3.6 eV^24^, which makes it transparent to visible light. This property makes SnO_2_ a desirable material for optoelectronic devices such as transparent conductive electrodes in solar cells and displays^22^. Apart from its optical characteristics, SnO_2_ exhibits remarkable electrical properties, including high electron mobility and low resistance, offering it a suitable candidate for employment in electronic domains^23,24^. SnO_2_ has also been explored as a potential material for resistive switching memory applications^25^. The reversible changes in resistance of SnO_2_ upon application of an electric field make it a suitable candidate for data storage and retrieval purposes. Hence, SnO_2_ is found to be an appealing contender for upcoming non-volatile memory equipment^26^.

Moreover, recent studies have indicated that SnO_2_ is capable of demonstrating threshold-switching phenomena^27^. The effect of threshold switching refers to the rapid transition of a material from a high resistance state (HRS) to a low resistance state (LRS) upon the attainment of a specific threshold voltage. This property exhibits significant potential for utilization in high-speed electronic devices.

Various techniques exist for the synthesis of thin films. The sol–gel technique is a common chemical wet deposition method utilized for the production of thin film oxide coatings, including TiO_2_ and SnO_2_^25,28–30^. In contrast to physical vapor deposition and chemical vapor deposition, the sol–gel method is commonly perceived as a more cost-effective and uncomplicated deposition technique, as it does not necessitate intricate vacuum systems or processing at elevated temperatures. Likewise, the sol–gel technique exhibits potential for industrial applications due to its ease of scalability for large-scale production.

In order to investigate the behavior of memristivity under the influence of light and humidity, it is imperative to consider the following traits. Firstly, positive photoconductance (PPC) and negative photoconductance (NPC) refer to the increase or decrease in the conductivity of a device upon exposure to light^31^. As an example of the NPC effect, Sheykhifard et al. investigated how the memristivity of 2D materials like graphene (or MoS_2_) can be tuned by illumination of the light and its effect on I_on_/I_off_ ratio^27^.

The resistive switching effect in memristors can be combined with PPC, leading to the emergence of innovative capabilities, such as optically programmable resistive switching. Utilization of this methodology can facilitate optical manipulation of the device's resistance, rendering it an attractive option for optical computing and neuromorphic systems^32,33^. Secondly, the negative differential resistance (NDR) effect is characterized by an atypical deviation in the “on-state” of the I–V curve, exhibiting a different behavior from that of conventional resistive components^34^. The existence of NDR in these devices can be attributed to the quantum mechanical effect of electron tunneling^32^. Upon application of a voltage to the device, electrons can tunnel through a thin barrier in the semiconductor material, thereby facilitating the flow of electric current. Within specific voltage ranges, tunneling exhibits enhanced conductivity resulting in a reduction of resistance and a concomitant augmentation of current flow. However, at higher voltages, the tunneling effect becomes less efficient, leading to an increase in resistance and a decrease in current flow^33^. Recently, the existence of both memristance and NDR effect in Gr/MoS_2_ heterostructures was reported as an interface-induced effect^35^. The coexistence of PPC and NDR effects with resistive switching (RS) memory behavior in certain materials is advantageous for attaining advanced simulations of biomimetic or neuromorphic computing^36^. This field of study endeavors to create computer systems capable of executing tasks similar to what happens in the human brain. The stability of synaptic weights is crucial for precise and dependable computation in a neuromorphic computing system^37^. Hence, attention is paid to employ materials that possess characteristics comparable to those of neural synapses, which encompass the capacity to store and process data^36^.

The present study points out that the implementation of Ag/SnO_2_/FTO structure results in the manifestation of memristive behavior in SnO_2_, thereby enabling its application as a switching resistor. Furthermore, this particular configuration exhibits photosensitivity and indicates a PPC phenomenon, whereby its conductivity is enhanced, particularly under ultraviolet (UV) irradiation. Furthermore, the NDR effect is demonstrated in the memristor under humid conditions. The concurrent manifestation of the NDR phenomenon and resistive switching (RS) memory characteristics are highly advantageous for applications in synaptic and neuromorphic computing, as evidenced in ref^37^.

## Experiment

Thin SnO_2_ layers were fabricated using an airbrush spraying technique. To produce the precursor solution, ZnCl_2_. 2H_2_O (Merck) was dissolved in isopropanol (extra pure) C_3_H_8_O and stirred for 1 h at ambient temperature. A stable uniform colloidal solution was made. While stirring, a certain volume of deionized water was introduced drop by drop to this solution for the hydrolysis process. The molar proportions of zinc chloride dihydrate, isopropanol, and water were 1:113:38. After 24 hours of stabilization at room temperature and in ambient air, the hydrolysis reaction was complete. This procedure yielded a transparent solution that is used as a precursor to the deposition of SnO_2_ thin film.

**Figure 1:**
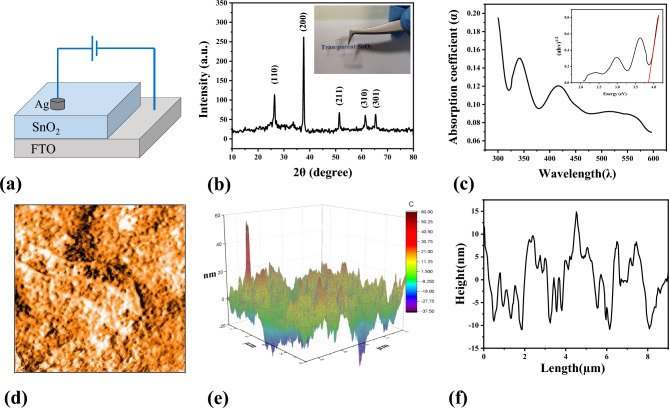
(**a**) A schematic diagram of an Ag(20–25 µm)/SnO_2_(~ 125 nm)/FTO(~ 300 nm) structure. (**b**) X-ray diffraction pattern of SnO_2_ sample, the inner part is a photo of the sample which shows its transparency (**c**) UV absorption spectra of SnO_2_, (**d**) AFM image of the surface of SnO_2_ thin film (9 µm × 9 µm), (**e**) 3D AFM image of SnO_2_, (**f**) The line scan of an AFM image.

The FTO glasses were purchased from the market and cut to a size of 1 × 1 cm. They underwent a preliminary cleaning process that consisted of acetone and ethanol in an ultrasonic bath. The FTO glass was placed on a hot plate at 240 °C for several minutes, and the prepared solution was sprayed onto it from a distance of 6 cm. SnO_2_/FTO glass was fabricated, and Ag paste was applied to specific areas of the sample in order to form contacts. The I–V measurements were utilized via a source meter (Keithley 2450). The schematic diagram of an Ag/SnO_2_/ FTO structure is illustrated in Fig. 1a. Regarding the thickness of the top electrode the PPC and the NDR effect, there exists a correlation between electrode thickness, threshold voltage, and forming voltage in the NDR^38^ and the photocurrent^39^. we considered that thickness remains unchanged and the FTO thickness is ~ 300 nm, the thickness of the SnO_2_ layer which is deposited by the airbrush is obtained around 125 nm, and the thickness of the Ag electrode which is used by Ag paste obtained around 20–25 µm. The obtained transparent sample is shown in Fig. 1b.

The impact of light on the sample was examined by subjecting the structure to light and measurements of electrical resistance and conductivity were recorded. The study revealed that the sample exhibits light sensitivity and the PPC effect has been detected. In order to examine the impact of humidity on the NDR phenomenon, the sample was placed in a container with an open side and the moisture was applied by a humidifier. The current–voltage (I–V) characteristics were obtained and subsequently evaluated for three discrete levels of humidity, specifically 95%, 70%, and 40%. The NDR phenomenon is distinguished by a discernible peak in the current–voltage (I–V) curve as a fingerprint of this effect.

In order to characterize some features like the crystal structure, morphology of the surface, and interaction of light with the sample, the following characterizations were performed. X-ray diffraction (XRD) was used to investigate the crystal structure of the sample (RIGAKU-Geigerflex diffractometer). The intensity of XRD was attained by changing 2θ from 10 to 80 degrees. Also, the morphology of the surface obtained by an atomic force microscope (Nanosurf Mobile-S portable) and the roughness parameters were investigated. The observation was attained in a window of 9 × 9 µm^2^ from the sample. In addition, according to the UV–visible measurements (Perkin Elmer Lambda 25 UV/vis spectrometer), SnO_2_’s absorption spectrum has been acquired and its band gap has been measured.

## Characterization

XRD pattern of tin dioxide thin films prepared by sol–gel technique is shown in Fig. 1b. The diffraction pattern of SnO_2_ exhibits peaks at (110), (200), (211), (310), and (301) with corresponding 2θ diffraction angles of 26.52°, 37.74°, 51.41°, 61.55°, and 65.37°, respectively. These peaks belong to the cassiterite crystal phase with a tetragonal rutile structure (JPDS No. 01-077-0452).

According to the UV–visible absorption spectrum of SnO_2_ at a wavelength of about 340 nm, which originates from the band gap transitions and directs the existence of highly crystalline SnO_2_, the maximum absorption occurred (Fig. 1c). Based on Davis-Mott and Tauc^40,41^, the absorption coefficient α is measured as a function of photon energy hν, and the data is plotted as (αhν)^2^ in terms of hν. The plot of (αhν)^2^ in terms of hν produces a straight line with a slope proportional to the absorption coefficient and an intercept proportional to the band gap energy E_g_. The equation for this line is given by (αhν)^2^ = B(hν − E_g_)^γ^, where h is Planck's constant, ν is the photon's frequency, and B is a constant. Depending on the nature of the electron transition, the value of γ is either 1/2 or 2, for direct and indirect transition band gaps, respectively. To determine the band gap energy E_g_, the straight lines of the spectra are extrapolated to the point where (αhν)^2^ = 0. The intersection of these lines with the energy-axis gives the value of hν at E_g_. According to Fig. 1c, the band gap energy E_g_ = 3.6 eV. The determination of the band gap energy is a crucial step in constructing a free band diagram of the structure to acquire a more comprehensive comprehension of the movements of charge carriers. Further elaboration will be provided subsequently.

AFM measurements have been performed to examine the surface of the film, giving detailed information on the nanoscale texture and film roughness. Figure 1d shows the non-uniform distribution of surface height of the thin film, where the lighter areas show the peaks and the darker areas represent the valleys. For a better understanding of this nonuniformity, a three-dimensional image of the surface height distribution is illustrated in Fig. 1e which shows a highly rough surface and spatial variations are clearly visible. We performed AFM line scan measurement diagonally along the resulting image (Fig. 1f). The R_q_ is the amount of the average deviation from the mean of the surface height and is commonly used to quantify the roughness of a surface. This R_q_ is attained between ~ 7 - 10 nm in a 9 × 9 µm^2^ window of the sample.

As evidenced by Fig. 1e, the surface exhibits a high degree of roughness, resulting in an increased effective surface area. This, in turn, facilitates greater charge accumulation, particularly in areas of sharp curvature. The presence of roughness is a significant factor in both the formation and disruption of conductive filaments. It is noteworthy that these sharp regions serve as the primary catalyst for the creation of conductive pathways.

## Result and discussion

The current–voltage (I–V) characteristics were examined both in the presence and absence of light to study the impact of light on the sample. These tests revealed the PPC effect as an indication of an increase in conductivity. Additionally, these measurements were performed while varying the available humidity exposed to the sample in order to investigate the NDR impact. The I–V curve for this effect is asymmetric, and there is an additional peak that results from an additional redox reaction emanating from available humidity. We will discuss our results in continuing.

### Mechanisms of photoconductance (the PPC and NPC effect)

The Ag/SnO_2_/FTO structure is a light-sensitive photo-memristor. When the sample is exposed to white light, the excitons produced by the light can change the conductivity in two ways known as negative photoconductance (NPC) and positive photoconductance (PPC). The NPC, or non-photoconductive state, is characterized by the inhibition of charge carrier participation in current conduction due to light emission, leading to an increase in resistance. Charge confinement occurs within the interfacial region of two adjacent layers, leading to a reduction in conductivity. Also, Zhou et al. reported that the NPC effect is attributed to the compensation of the oxygen vacancies, the restriction of injected electrons, and the variation in the electrostatic potential^42^.

PPC, or positive photoconductance effect, is a phenomenon in which the absorption of light induces the flow of charge carriers, resulting in a decrease in resistance and an increase in current conduction.

**Figure 2:**
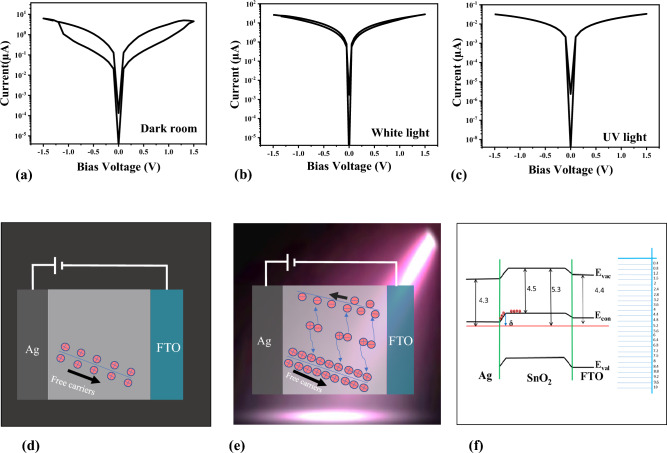
(**a**) I–V curve of structure by exposing the sample in the darkness, (**b**) white light, (**c**) UV light. (**d**) SET process under low bias and in the absence of light. (**e**) SET process under low bias and presence of UV light. (**f**) Diagram band energy of the memristor. Where (${\upvarphi }_{{{\text{SnO}}_{2} }} = - 5.3\;{\text{eV}}$, ${\upvarphi }_{{{\text{FTO}}}} = - 4.4\;{\text{eV}}$, ${\upvarphi }_{{{\text{Ag}}}} = - 4.3\;{\text{eV}},\;{\text{E}}_{{{\text{g}}\;{\text{SnO}}_{2} }} = 3.6\;{\text{eV}}$, ${\text{E}}_{{{\text{g}}\;{\text{FTO}}}} = 3.65\;{\text{eV}}$).

Excitons are separated and generate charge carriers at the Ag and SnO_2_ interface in our structure. The existence of a trap located at the heterogeneous interface of two distinct materials leads to the separation of excitons and the birth of hot carriers, thereby augmenting conductivity. The memristivity of the sample is illustrated in Fig. 2 under two conditions: with and without the presence of light. Figure 2a depicts the current–voltage (I–V) measurement in the absence of light, whereas the I–V measurement in the presence of white light and UV emission is depicted in Fig. 2b,c, respectively. In a dark environment, at a certain voltage (1 V) the current that passes in the “on” state is five times greater than of the “off” state i.e. $\left( {\frac{{{\text{I}}_{{{\text{on}}}} }}{{{\text{I}}_{{{\text{off}}}} }} = 5.4} \right)$ (from 0.002 to 0.00037 mA). The ratio $\frac{{{\text{I}}_{{{\text{on}}}} }}{{{\text{I}}_{{{\text{off}}}} }} = 1.12$ decreases under light illumination and by applying UV light this ratio almost vanished. It is clear that white light and UV emission increased the conductivity, confirming the PPC effect. It is noted that the measurements for UV illumination was carried out with a larger probe distance than what is done for white light illumination.

There are two preferable states in our experiment; initial state (dark condition) and exposing sample to a source of light. First, the initial state (dark conditions).

In the absence of light (Fig. 2d), there is a relatively long Schottky barrier between Ag electrode and SnO_2_. When a small positive bias voltage is applied, most of the free carriers are trapped at the Ag/SnO_2_ interface and only a small number of them can cross the barrier and the loop in I–V curve hysteresis is considerably wide (Fig. 2a(. So, in this case, the equilibrium state is present and the initial thermal-equilibrium conductivity can simply obtained by $\sigma_{0} = e\left( {\mu_{n} n_{0} + \mu_{p} p_{0} } \right)$ which $\mu_{n}$ is electron mobility, $\mu_{p}$ is hole mobility $n_{0}$ is concentration of electrons and $p_{0}$ is the concentration of holes.

By exposing the sample to the light, the movement of electrons is facilitated due to the smaller Schottky barrier that electrons face between the Ag electrode and SnO_2_. So, the loop in I–V curve become narrower (Fig. 2b). In this case, by applying light, excitons are generated in the presence of an external electric field and subsequently separate at the interface, as illustrated in (Fig. 2e(. If excess carriers are generated in the semiconductor, the conductivity becomes $\sigma_{0} = e\left( {\mu_{n} (n_{0} + \delta n} \right) + \mu_{p} \left( {p_{0} + \delta p} \right))$. where $\delta n$ and $\delta p$ are the excess electron and hole concentrations, respectively. By considering n-type SnO_2_ the concentration of electrons and holes become equal so $\delta n = \delta p$ and by exposure to light (Fig. 2e), the change in conductivity due to the optical excitation, known as the photoconductivity, is the $\Delta\upsigma = {\text{e}}\left( {\updelta {\text{p}}} \right)\left( {\mu_{n} + \mu_{p} } \right)$. In this case, the total current density can be written as $J = J_{0} + J_{L} = (\sigma_{0} + \Delta \sigma )E$. The generation rate of excess carriers by exposing to the light or UV radiation, ${\text{G}}_{{\text{L}}}$ can be written as^43^

1 $${\text{G}}_{{\text{L}}} = \frac{{{\text{I}}_{{\text{L}}} }}{{e\left( {\frac{{\tau_{p} }}{{t_{n} }}} \right)\left( {1 + \frac{{\mu_{p} }}{{\mu_{n} }}} \right){\text{AL}}}}$$

where ${\text{I}}_{{\text{L}}}$ is photo-current $\tau_{p}$ is excess minority carrier lifetime, $t_{n}$ is electron transit time, A is the effective surface area and L is the length of sample. According to Eq. (1), the generation of hot carriers is reversely proportional to the length of sample by increasing the length the generation of hot carriers decreases.

Photons can modify the Schottky barrier and band structure of this sample. By considering these values for work functions (φ) and band gap energies (E_g_), the heterojunction band diagrams with two interfaces can be shown as (Fig. 2f). Photons affect the electrical characteristics of SnO_2_. The Schottky barrier at the Ag/SnO_2_ interface is significantly altered. Electrons must cross the Schottky barrier to move from semiconductor to metal. This alteration in the energy band's curvature and the Schottky barrier's height (δ) allows carriers to more easily overcome the potential barrier and increases conductivity. Light absorption excites SnO_2_ electrons from the valence band to the conduction band. This creates electron–hole pairs. Importantly, the interface-generated carriers face a reduced Schottky barrier. This reduced barrier helps electrons pass from the semiconductor to the metal electrode. Therefore, the carriers exhibit enhanced mobility and can more easily traverse the barrier.

In the present state, the carriers exhibit easy traversing through the barrier, resulting in an increase in conductivity and a narrower hysteresis loop in the I–V curve. The UV–visible spectrum analysis (Fig. 1c) has revealed that SnO_2_ exhibits an absorption peak at a wavelength of 340 nm, which falls within the UV light range. It derives from the band gap transitions and indicates to the presence of crystalline SnO_2_. So, it can be recognized that by applying the UV light, the PPC effect occurs and it raises up the conductivity, which is in conformity with our result represented in (Fig. 2c). The intensification of the PPC effect and the near disappearance of the loop in the I–V curve can be attributed to the close proximity of the wavelength of UV emission and the band gap of SnO_2_.

### The coexistence of the NDR effect and RS behavior

In this work, both NDR and RS properties observed in the Ag/SnO_2_/FTO structure by applying a low bias voltage. These effects were elicited by exposing the sample to moisture produced by a humidifier, concurrently with the application of a low bias voltage. Ji et al. have achieved that the coexistence of NDR effect and RS memory behavior can be modulated by the moisture with a good reversibility at room temperature^44^.

The observation of the NDR effect is dependent on the ratio of water molecules found in the surrounding atmosphere. This phenomenon is inherent in normal conditions and can be enhanced through the introduction of additional humidity. In the typical ambient environment and at normal room temperature, the process of adsorption occurs where gaseous water molecules adhere to the surface of the structure. Following this, the water molecules then interact with the oxygen that is present within the lattice structure, as well as any oxygen vacancies that may be present and then produces hydroxide ions to migrate to SnO_2_ bulk. Valov and co-workers have pointed out that water molecules are easily absorbed in a thin layer with a nano porous structure^45,46^. In electrochemical concepts, the peaks observed in the plot serve as indicators of the redox reaction occurring within the sample. So, by applying extra humidity one can manipulate oxidation and reduction reactions and intensify the NDR effect^36^. Similar to our results, the NDR effect has been reported earlier in many switching devices with different reasons such as charge trapping and detrapping^47^ and ion migration^48,49^.

A significant NDR peak was observed when the sample was subjected to 95% humidity and the corresponding current–voltage (I–V) curve was measured simultaneously. Then, the source of the humidifier was turned off, and over time, the amount of humidity that was accessible within the container reduced. This measurement was repeated at 70% humidity after five minutes and 40% humidity after twenty.

**Figure 3:**
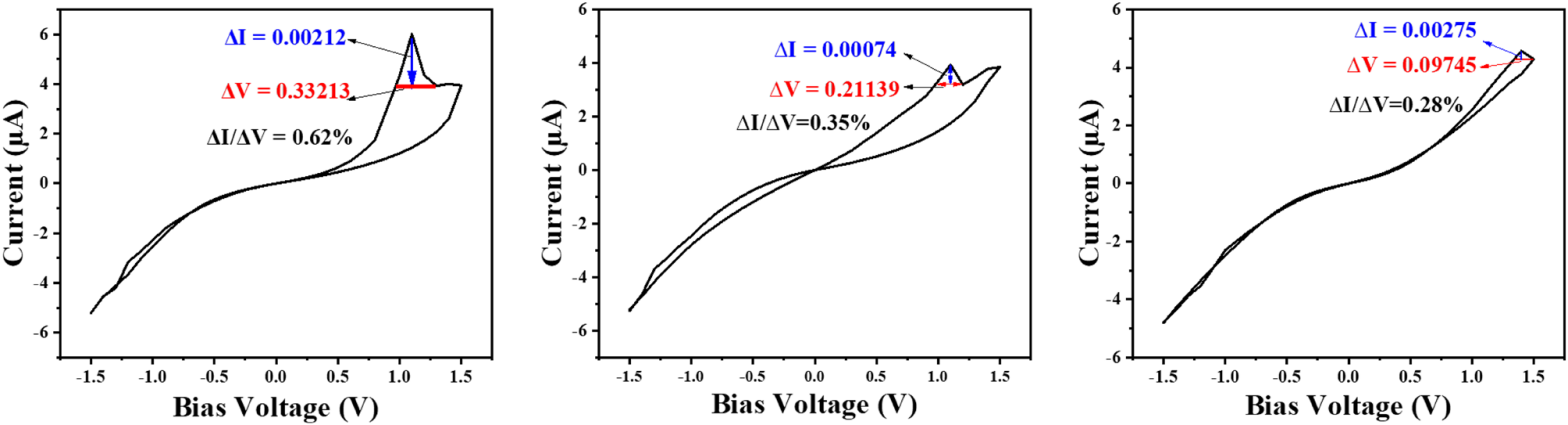
Current–voltage (I–V) curve in presence of (**a**), 95% moisture. (**b**) 70% moisture (after 5 min), (**c**) 40% moisture (after 20 min).

The memristive characteristic, particularly observed in metal oxides, is influenced by the oxygen concentration within the surrounding environment. As depicted in Fig. 3, the passage of time leads to a reduction in humidity as a result of water molecule absorption by the structure, consequently leading to a decrease in atmospheric oxygen levels. The reduction of humidity from 95 to 40% resulted in a decrease in the NDR peak. However, as depicted in Fig. 3b,c, it is evident that the conductivity exhibited an increase over time. This can be attributed to the penetration of water molecules into the structure. The sharpness of the NDR peak at 95% relative humidity is roughly twofold greater than when the relative humidity is 70%. The relativity of ∆I/∆V reduced in 0.35% by decreasing the humidity from 95 to 70% and this value reached 0.28% with 40% of humidity. We note that the memristivity degrades with successive cycling that requires a deep investigation.

As previously discussed, there is a notable surface roughness that contributes to an increased effective surface area. This enhanced surface area facilitates the acceleration of the redox reaction and enables the preservation of conductive pathways. Although the tin dioxide developed in this study has a tetragonal structure, its polycrystalline structure and abundant grain boundaries with oxygen vacancies may provide ion localization.

The presence of oxygen facilitates the migration of metal ions, which is necessary for the formation of these filaments^36^. The presence of oxygen vacancies in memristivity holds significant importance and plays a pivotal role, alongside various other factors including temperature, voltage, and material composition, in determining the behavior of these devices.

**Figure 4:**
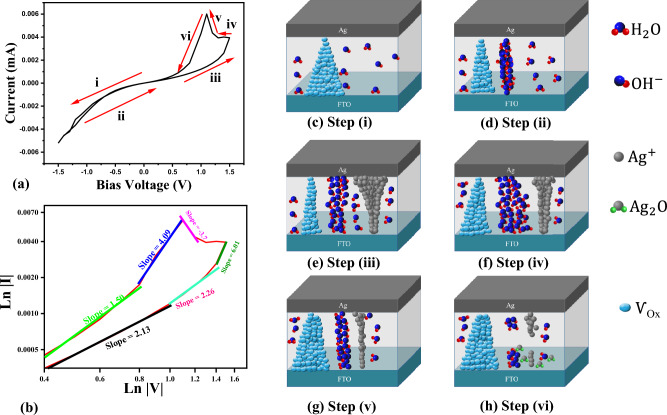
(**a**) Current–voltage (I–V) curve of Ag/SnO_2_/FTO under 0.95% relative humidity and voltage scan rate of 1000 mVS^−1^. (**b**) Double-log I–V curves and fitting results of SnO_2_ memristor. (**c**–**h**) Physical mechanism of memristivity in the presence of humidity.

In order to elucidate the phenomenon of NDR, the current–voltage (I–V) characteristic curve was partitioned into six distinct operational stages ((i) to (vi) stages in Fig. 4 relative to Fig. 4c–h). Water molecules provide ${\text{OH}}^{ - }$ ions, which have the ability to influence redox reactions during the I–V measurements. The absorbed water molecules in the reaction with oxygen inside the semiconductor as well as oxygen vacancies (Ѵ_Ox_) result in the formation of hydroxide ions (${\text{OH}}^{ - }$) as described by Eq. (2). Also, high effective surface area of SnO_2_ and abundant grain boundaries with provision of oxygen vacancies produce ${\text{OH}}^{ - }$. In step (i), according to the less ${\text{OH}}^{ - }$ migration at low bias voltage, memristor is still in HRS mode.2

This equation is known as hydric reaction^40^. Where ${\text{O}}_{0}^{{\text{x }}}$ and Ѵ_Ox_ denote the oxygen in the lattice and oxygen vacancies, respectively. By changing the voltage from 0 to − 1.5 V, a large volume of Ѵ_Ox_ migrates toward the Ag electrode, which causes the formation of a conductive filament of Ѵ_Ox_ between the Ag and FTO electrodes. Afterward, the memristor switches into LRS mode (SET process).

In step (ii), as the voltage changes from − 1.5 V to 0, the memristor remains in the LRS until the slope of the I–V curve experiences a significant decrease as illustrated in Fig. 4d and associated with a voltage of − 0.6 V in Fig. 4a. Then according to the slope of the I–V curve, the memristor is placed in a HRS. The observed rise in resistance can be attributed to a concurrent reduction in the electric field. At this stage, regardless of the restriction imposed by the negative bias voltage on the migration of ${\text{OH}}^{ - }$ ions, these ions actively contribute to the electrical conductivity and enhance the current.

In step (iii), when the voltage increases from 0 to 1.5 V, the migration of Ag^+^ ions towards the FTO electrode is facilitated by positive bias voltages and reduced by an electron to form the Ag as stated in Eq. (3). By increasing the electric field, the Ag^+^ ions accumulate at the interface of FTO, eventually forming a conductive filament. Additionally, the presence of a positive bias at the interface of the FTO electrode encourages the attraction of ${\text{OH}}^{ - }$ ions. Hence, this process facilitates the formation of an ${\text{OH}}^{ - }$ conductive pathway, ultimately leading to an increase in the current. At the voltage of 1.5, the simultaneous presence of two Ag and ${\text{OH}}^{ - }$ pathways are responsible for the conductivity of the sample. However, the filament of the oxygen vacancies is also weakly present^45^.

3 $${\text{Ag}} \rightleftarrows {\text{Ag}}^{{ + { }}} + {\text{e}}^{ - }$$

Plus, during this particular stage, the oxygen vacancies progressively migrate back to the FTO electrode, thereby signifying a smooth relaxation of the Ѵ_Ox_ filament.

In step (iv), the reduction of voltage from + 1.5 V to 0 results in a decrease in positive bias, which weakens the Ag filament. Over time, the ${\text{OH}}^{ - }$ ions find an opportune moment to enhance their strength and according to Eq (2) starts to stablish a conductive path of oxygen vacancies. And by some means, these filaments are in the trade-off, and regarding the competition between them the current may remain intact. Also, it may face a decrease and appear as a deep in the I–V curve. (Here the current remained unchanged until the threshold voltage “V_th_” was reached V_th_ = 1.3 V (see Fig. 4a-step iv, and Fig. 4d).

In stage (v), combined with the argument presented in the step prior, at the threshold voltage (V_th_), alongside the presence of dwindled silver and the ${\text{OH}}^{ - }$ filament, the oxygen vacancy filaments formed with the highest quantity resulting in a peak in current despite the reduction of voltage. As mentioned by Xiaofang Hu et al.^40^ the presence of these three filaments is responsible for the appearance of this peak, which induces the system to switch into the LRS (see Fig. 4a-step v, and Fig. 4g).

The Ag^+^ ions then migrate to the FTO. In this stage, oxidation and electron loss take place (see Eq. 4). These electrons also contribute to the generation of peak current. In fact, at this juncture, two constituents of the conductive filament, ${\text{OH}}^{ - }$ and Ag^+^, as well as electrons expelled by oxidation reactions, contribute to the generation of current^46^.

4 $$2{\text{H}}_{2} {\text{O}} \leftrightarrow {\text{O}}_{{2{ }}} + 4{\text{H}}^{ + } + 4e^{ - } { }$$

In stage (vi), through the progressive reduction of voltage and decrease in the applied field, the driving force of ${\text{OH}}^{ - }$ and Ag^+^ is weakened and both ${\text{OH}}^{ - }$ and Ag^+^ filaments are reduced until both filaments are ruptured and the value of current becomes zero. While, in this stage, the filament of oxygen vacancy has the highest concentration until the available water splitting decreases due to a drop in voltage and therefore the reduction in available ${\text{OH}}^{ - }$ which is responsible for the intensification of oxygen vacancies and the system is managed by HRS.

It worth to mention that an unstable AgOH is formed (Eq. 5) due to the reaction of ${\text{OH}}^{ - }$ and Ag^+^ and immediately decomposes to Ag_2_O at room temperature. At this time, the ion migration in the body and surface of SnO_2_ is limited by Ag_2_O. As a limitation of this structure, it should be mentioned that there is an undesirable reaction that leads to producing silver oxide. The current limitation by Ag_2_O is also weak. Rupturing and tearing the conductive filaments cause the device to return to HRS mode (Eq. 6).

5 $${\text{Ag}}^{ + } + {\text{OH}}^{ - } \leftrightarrow {\text{ AgOH}}$$

6 $$2{\text{AgOH}}^{ - } \leftrightarrow {\text{ Ag}}_{2} {\text{O}} + {\text{H}}_{2} {\text{O}}$$

By analyzing oxygen vacancies and their function in the VCM process, our study recognizes the importance of trap states in the bandgap, which capture carriers and change SnO_2_ conductivity in response to light and moisture. The mechanism that is related to oxygen vacancy is VCM which can appear in two types filamentary and interfacial and the accumulation of them leads to a conductive path. If we consider the minimum number of oxygen vacancies that can form a conductive path (N), and each oxygen vacancy is represented by (n) then we can say $N = \sum\nolimits_{1}^{N} {n_{i} }$. The N parameter is related to many factors like Electric field, thickness of layers, roughness parameters of layers, contact area, light intensity, moisture intensity, and electrode distance should be included in a comprehensive analysis and a statistical or semi-analytical approach is needed to delve deeper how all these affect on this mechanism.

#### Fitting the current–voltage curves with the space charge limited current (SCLC) model

To describe charge transport in thin-film devices, such as memristors, SCLC model is commonly used. This model can be described by Eq. (7)^41,50^

7 $${\text{J}} \propto \frac{{{\text{V}}^{{{\text{m}} + 1}} }}{{{\text{d}}^{{2{\text{m}} + 1}} }}$$

where J is the current density, V is the bias voltage in units of Volt, d is the thickness of the tin dioxide in units of nm and m is the fitting index which is a dimensionless constant parameter.

The SCLC model contains three regimes that describe the (I–V) characteristics of the device. (i) Low voltage Ohmic region which is described by Ohm's law, J = GV, (ii) Child's square law^51^ region which J ∝ V^2^ and (iii) Current steep increase region which J ∝ V^α^, where α represents the order of voltage between 3 and 4. At a relatively low bias voltage region, (m = 0), the current density is dominated by Ohm's law and it is proportional to the applied voltage. In a fairly high bias voltage region, (m = 1), the current density is dominated by Child's law. So, the current density increases with the square of the applied voltage and can be described by J ∝ V^2^. To explain this region, double-log I–V curves in the positive voltage region are replotted in Fig. 4b. By obtaining the slopes of different regions in HRS mode, the fitting value of 2.13 and 2.26 imply that the Child's law (i.e., the oxygen vacancy migration) may dominate the charge behaviors in this region. In LRS mode at the low bias, the fitting value of 1.5 indicates that the Ohmic conduction^41^ (i.e., the Ag metallic filaments) is dominated. In high bias voltage region (both in HRS and LRS part) the fitting value of 3.7, 4.09 and 6.01 indicate the Schottky tunneling mechanism. The Schottky tunneling model^50,52–54^ can be described by Eq. (8) and (9)

8 $$J \propto \exp \left( {\frac{\beta }{{k_{B} T}}E^{\frac{1}{2}} - \frac{\varphi }{{k_{B} T}}} \right)$$

9 $$\beta = \sqrt {\frac{{q^{3} }}{{k\pi \varepsilon_{0} \varepsilon_{r } }}}$$

That $\varphi$, $k_{B}$*,* T, E, q, $\varepsilon_{0}$ and $\varepsilon_{r }$ denotes the Schottky barrier, Boltzmann constant, temperature, electric field, electric charge , vacuum permittivity and relative permittivity, respectively.

### Stability

As confirmed by Xiaofang Hu and his colleagues^40^, the coexistence of NDR and RS in a memristor improves storage density and reduces power consumption. Also, X. Ji and his colleagues^44^, reported that the coexistence of these two effects can change memory time without additional circuitry and cost.

One of the important characteristics of memristors is stability, which refers to their ability to maintain the resistance state over time. Also, stability has a key role in the development of reliable and efficient neuromorphic computing systems. Some studies have shown that certain types of memristors, such as those made from oxide materials, can exhibit stable resistance states over long periods of time^55^.

**Figure 5:**
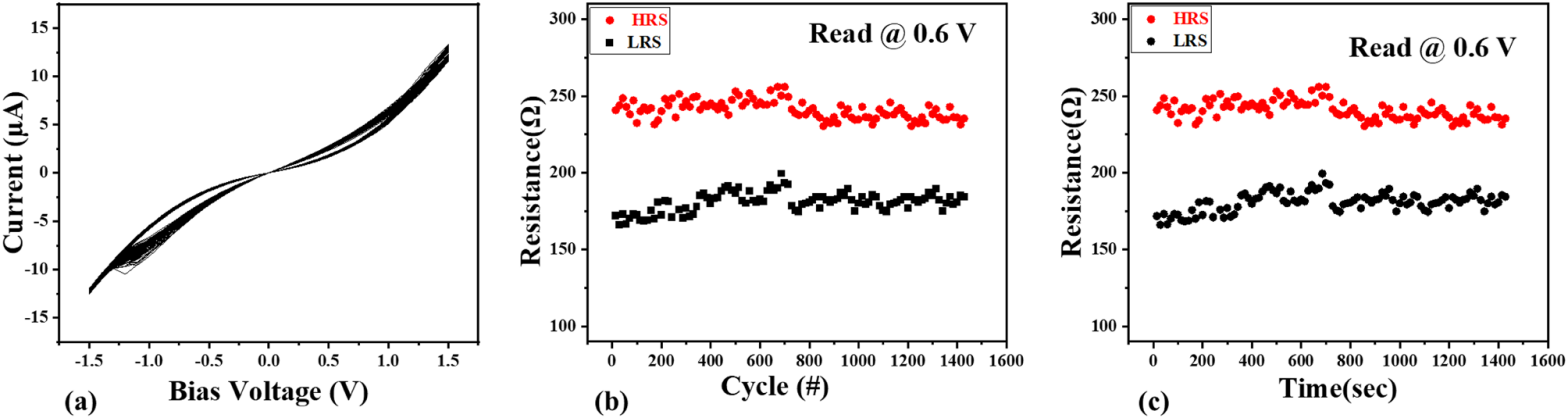
(**a**) Reliability and randomness of memristors. (**b**) Endurance of HRS and LRS at a reading voltage of 0.6 V showing remarkable stability. (**c**) Retention characteristics of the Ag/SnO_2_/FTO structure at 0.6 V read voltage.

The observed sensible stability of the memristor in this particular structure renders it a dependable choice for applications in memory and neuromorphic computing. Figure 5a exhibits the remarkable stability of the SnO_2_ memristor throughout 100 cycles. Additionally, Fig. 5b showcases the endurance of the HRS and LRS over 100 voltage cycles, demonstrating exceptional repeatability. As shown in Fig. 5c, our sample has reasonable stability in the ambient temperature for about 1500 s and the memory window does not change or deviate significantly, but the LRS and HRS level difference in the initial memristor operation is 70 units. After 375 s (25 cycles), this amount drops to 56 units. Due to the incomplete formation of CFs such Ѵ_Ox_, ${\text{OH}}^{ - }$ and Ag^+^, the structure has lower resistance and higher conductivity.

CFs strengthen and conductivity increases over time, decreasing the LRS-HRS level differential and memory window. From 800 s onward, the memory window remained steady, indicating that from 800 to 1500 s, CFs are competing with each other, but their results have not changed the memristivity and memory window.

The performance comparison of memory devices developed with different electrolyte layers is summarized in Table 1. These findings lead to the important fundamental insight that the type of material of the switching layer as well as the amount of oxygen vacancies play a very important role in the performance and improvement of memristor efficiency.

**Table 1:** Performance comparison of SnO_2_-based memory and other memory devices.

References	Metristor model	Switching type	I_LRS_/I_HRS_ ratio	Endurance (cycles)	Retention time (s)
^1^	Au/Cu_x_O/ITO	RS	~ 10^4^	200	10^4^
^4^	Ag/GQDs/TiO_x_/FTO	RS + NPC	> 1.5 × 10^2^	300	10^4^
^6^	Ti/Pt/SnO_2_-QDs/Pd	RS	~ 10^3^	110	N.S
^14^	Ag/HfO/ITO	RS + TS	~ 10^2^	100	10^3^
^25^	ITO/SnO_x_/HfO_x_/ITO	RS + LS	~ 10^2^	10^4^	> 10^4^
^41^	Ag/AZB:PMMA/FTO	RS	~ 10^4^	100	N.S
^43^	Ag/TiO_x_/FTO	RS + NDR	~ 55	1200	10^4^
^46^	Ag/NPs-TiO_x/_FTO	RS + NDR	N.S	200	> 10^3^
^55^	Al/Sb:SnO_2_/TiO_2_/Al	RS	> 8 × 10^2^	100	> 10^3^
This work	Ag/SnO_2_/FTO	RS + NDR + PPC	> 5.4	100	> 1.4 × 10^3^

*TS* Tunneling switching, *PPC* Positive photoconductance, *NDR* Negative differential resistance, *N.S* Not stated.

There are some effective parameters and strategies which is mentioned below to achieve a larger memory window. Using materials with higher work function as a bottom electrode, (e.g., Pt), and active materials with a higher electronegativity for the top electrode (TE), (e.g., Au), is recommended. Also, using some 2D materials is advised for TE for instance Graphene oxide (GO). GO has a high surface area, which can provide a large contact area with the SnO_2_ layer. This increased contact area can facilitate more reliable charge transport and switching behavior. Furthermore, the choice of electrode configuration (e.g., planar, vertical, or crossbar) can also impact the memory window which may provide better control over the switching process. Additionally, choosing a switching layer with less thickness may improve the memory window. The quantum effects can show themselves better in down scales. As another suggestion, surface modifications (e.g., the treatment of oxygen plasma) or adding interlayers at the electrode-semiconductor interface can reduce defects and enhance charge carrier injection. Also, use high-quality, low-defect SnO_2_ films to improve the overall device performance.

## Conclusion

A memristor with a structure of Ag/SnO_2_/FTO exhibits the coexistence of NDR and RS behavior at room temperature. We demonstrated this memristive property for the first time in this structure. This structure can be used as a photo-memristor and when it is exposed to light, especially UV light, its conductivity increases, which confirms the existence of the PPC effect. Also, the NDR effect was observed due to the rupture or resurgence of metallic, oxygen vacancy, or hydroxide (${\text{OH}}^{ - }$) conductive filaments. These results can be beneficial for neuromorphic applications and its stability over 100 cycles enables it to maintain their synaptic weights over time which is important in the strength of the connection between two neurons.

## Data Availability

The datasets used and analyzed during the current study are available from the corresponding author on reasonable request.
